# Inter- and intra-observer agreement of the AO classification for operatively treated distal radius fractures

**DOI:** 10.1007/s11751-015-0237-z

**Published:** 2015-11-27

**Authors:** Jesse M. van Buijtenen, Mischa L. C. van Tunen, Wietse P. Zuidema, Emile A. Heilbron, Jeroen de Haan, Henrica C. W. de Vet, Robert J. Derksen

**Affiliations:** Department of Surgery, VU University Medical Centre, 1007 MB Amsterdam, The Netherlands; Department of Radiology, VU University Medical Centre, Amsterdam, The Netherlands; Department of Surgery, Westfriesgasthuis, Hoorn, The Netherlands; Department of Epidemiology and Biostatistics and the EMGO Institute for Health and Care Research, VU University Medical Centre, Amsterdam, The Netherlands; Department of Surgery, Zaandam Medical Centre, Zaandam, The Netherlands

**Keywords:** Distal radius fracture, Surgical procedures, Intra-observer agreement, Inter-observer agreement, AO classification, C-type fractures

## Abstract

The reproducibility of the AO classification for distal radius fractures remains a topic of debate. Previous studies showed variable reproducibility results. Important treatment decisions depend on correct classification, especially in comminuted, intra-articular fractures. Therefore, reliable reproducibility results need to be undisputedly determined. Hence, the study objective was to assess inter- and intra-observer agreement of the AO classification for operatively treated distal radius fractures. A database of 54 radiographs of all AO types (A, B and C) and groups (A_2-3_, B_1-3_, and C_1-3_) of distal radius fractures was assessed in twofold. Likewise, a subset of 152 radiographs of solely C-type groups (C_1-3_) was assessed. All fractures were classified by six observers with different experience levels: three consultant trauma surgeons, one sixth-year trauma surgery resident, a consultant trauma radiologist, and an intern with limited experienced. The inter-observer agreement of both main types and groups was moderate (*κ* = 0.49 resp. *κ* = 0.48) in combination with a good intra-observer agreement (*κ* = 0.68 resp. *κ* = 0.70). The inter-observer agreement of the subset C-type fractures group was fair (*κ* = 0.27) with moderate intra-observer agreement (*κ* = 0.43). According to these results, the reproducibility of the AO classification of main types and groups of distal radius fractures based on conventional radiographs is insufficient (*κ* < 0.50), especially at group level of C-type fractures.

## Introduction

The lifetime risk of sustaining a distal radius fracture is 15 % for women and 2 % for men [1]. Through the years, many different classification systems were developed for distal radius fractures [2]. Nowadays, the most frequently used classification system is that of the Arbeitsgemeinschaft für Osteosynthesefragen (AO). This system, based on an alphanumeric system, was developed by Müller and colleagues in 1986 and was slightly modified in 1990 [3]. Starting point was that the classification needed to be logical and consistent, reflect fracture complexity, easy to reproduce, and internationally comprehensive making it eligible for data processing [4]. The correct classification in combination with the AO surgical reference tool may guide clinicians in decision-making with regard to the treatment of these fractures.

The AO system allocates a code to the fracture based on its location and morphology. Distal radius fractures are referred to as “AO-23” fractures, in which “2” means forearm and “3” stands for distal. As for morphology, the fracture is divided into three types: extra-articular (A), unicondylar or combined metaphyseal (B), and intra-articular fractures (C). Each fracture type is subdivided into three groups (1, 2, or 3) based on fracture location and fracture morphology (complexity of the fracture) [3–5].

Since the new millennium, the diagnostic performance of the system was investigated and yielded variable results. The inter- and intra-observer agreement of these studies varied from fair to good [6–9]. These results are not consistent and raise questions on clinical usefulness in daily practice.

Classification systems should have acceptable inter- and intra-observer agreement since reproducibility is a key clinimetric property of a diagnostic test. Differently classified fractures may lead to different treatment options resulting in suboptimal outcomes.

The need for this study was deemed clear due to the inconsistent reproducibility results from existing literature on the AO distal radius classification system. Therefore, the study objective was to assess inter- and intra-observer agreement of the AO classification for operatively treated distal radius fractures.

## Materials and methods

All consecutive patients between 18 and 60 years of age who had been operatively treated for a distal radius fracture between January 1, 2007 and December 31, 2010 were included in this study. Eligible patients were identified by cross-referencing hospital diagnostic codes. A database of 54 digitized radiographs of all types (A, B and C) of distal radius fractures could be constructed. Since C-type fractures are the most complex and unstable fractures, operative treatment is often necessary to stabilize the fracture. Therefore a group of 152 radiographs consisting of solely C-type fractures was assessed separately at group level (C_1-3_).

Sample-size estimation was based on the rule of thumb that 50–100 patients are needed in order to obtain adequate power for a study on reproducibility [10]. Exclusion criteria were bone abnormalities, previous distal radius fractures, isolated ulna fractures (AO A_1_), and incomplete radiograph series.

Both radiographs of acute distal radius fractures and radiographs directly after closed reduction were used since decision-making is largely based upon these two series. The observers were blinded for patient characteristics and for each other’s answers. All radiographs were assessed twice by six different observers: three consultant trauma surgeons (WZ, JH, RJD), a consultant trauma radiologist (EH), a sixth-year trauma surgery resident (JB), and an intern with limited experienced (MT). A handout depicting the AO classification was used during all assessments. Each of the observers classified the radiographs in the same order, independently and at their own pace. The observers assessed all radiographs twice; the second assessment was shuffled and repeated after 3 weeks to avoid recall bias. The overall group was analyzed both at the level of distinction between main types (A, B and C) and at group levels (A_2-3_, B_1-3_ and C_1-3_). The agreement of main types and groups was calculated using the data from the assessment of the overall classification.

Cohen’s Kappa and 95 % confidence interval were calculated to render inter- and intra-observer agreement. It was assumed that misclassifications between two categories close to each other are less severe (i.e., A_2_ vs. A_3_) than misclassifications between categories which are further apart (i.e., A_2_ vs. C_3_), and therefore a weighted kappa was used [11]. Quadratic weights were used since these are usually applied in these instances. Since the weighted quadratic kappa equals the intra-class correlation coefficient of agreement and intra-class correlation can be calculated for groups of observers, calculation of intra-class correlation coefficient was used to obtain a value for the group kappa coefficient [11].

The kappa coefficients of the inter- and intra-observer agreement were classified according to the Landis and Koch classification: *κ* = 0.00 ‘Poor’, 0–0.20 ‘Slight’, 0.21–0.40 ‘Fair’, 0.41–0.60 ‘Moderate’, 0.61–0.80 ‘Substantial’, and 0.81–1.00 ‘Near ‘perfect’ [12]. In general, kappa values of <0.5 are considered unsatisfactory [13]. The inter- and intra-observer agreement of the types and groups were assessed using SPSS v16.0 (IBM, Armonk, New York).

## Results

In total, 54 radiographs of all types (A, B and C) and groups (A_2-3_, B_1-3_ and C_1-3_) of operated distal radius fractures and 152 radiographs of exclusively C-type fractures (C_1-3_) were assessed in twofold.

### Inter-observer agreement

#### All types (ABC) and groups (A_2-3_, B_1-3_ and C_1-3_)

For all six observers, the mean Cohen’s kappa for both types and groups was moderate (*κ* = 0.49 and 0.48) (Table 1). As for the three consultant trauma surgeons in particular, the mean kappa coefficient of the main types and that of their groups were both fair (*κ* = 0.39).

**Table 1:** Inter-observer agreement

Classification	All observers			Trauma surgeons		
	*κ* First assessment (95 % CI)	*κ* Second assessment (95 % CI)	Mean *κ* value	*κ* First assessment (95 % CI)	*κ* Second assessment (95 % CI)	Mean *κ* value
Main AO types ABC (*N* = 54)	0.47 (0.35–0.60)	0.50 (0.38–0.63)	**0.49**	0.32 (0.16–0.50)	0.45 (0.28–0.60)	**0.39**
Groups A_2-3_, B_1-3_, C_1-3_ (*N* = 54)	0.46 (0.32–0.60)	0.50 (0.37–0.63)	**0.48**	0.32 (0.14–0.50)	0.47 (0.30 –0.62)	**0.39**
Group C_1-3_ (*N* = 152)	0.30 (0.21–0.40)	0.24 (0.14–0.34)	**0.27**	0.31 (0.21–0.42)	0.30 (0.13–0.46)	**0.31**

Kappa value and 95 % confidence interval for the inter-observer agreement of all observers and the trauma surgeons separately

#### C-type fractures (C_1-3_)

The kappa coefficient concerning all observers for the separate C- type fractures group was fair (*κ* = 0.27). In the consultant trauma surgeon group, the inter-observer agreement was fair (*κ* = 0.31).

### Intra-observer agreement

#### All types (ABC) and groups (A_2-3_, B_1-3_ and C_1-3_)

The kappa values of the intra-observer agreement for all main types and groups for all observers were both found to be good (*κ* = 0.68 and 0.70) (Table 2). For the three trauma surgeons, the mean kappa value of the main types was moderate (*κ* = 0.60) and at group level was good (*κ* = 0.63).

**Table 2:** Intra-observer agreement

	ABC	A_2-3_, B_1-3_, C_1-3_	C_1-3_
Assessor 1 resident	0.87 (0.79–0.92)	0.88 (0.80–0.93)	0.25 (0.09–0.39)
Assessor 2 intern	0.60 (0.40–0.75)	0.64 (0.45–0.77)	0.52 (0.39–0.62)
Assessor 3 radiologist	0.77 (0.63–0.86)	0.80 (0.67–0.86)	0.48 (0.35–0.59)
Assessor 4 trauma surgeon	0.54 (0.29–0.71)	0.56 (0.32–0.73)	0.47 (0.32–0.59)
Assessor 5 trauma surgeon	0.68 (0.49–0.80)	0.69 (0.49–0.81)	0.43 (0.29–0.55)
Assessor 6 trauma surgeon	0.60 (0.40–0.75)	0.65 (0.47–0.78)	0.45 (0.31–0.56)
Mean kappa value	0.68	0.70	0.43

Kappa value of intra-observer agreement and 95 % confidence interval for main groups (A–C), subgroups (A_2-3_, B_1-3_, C_1-3_) and type C fractures

#### C-type fractures (C_1-3_)

The mean kappa value for the intra-observer agreement of C-type fractures is both moderate for all observers (*κ* = 0.43) and the group of trauma surgeons (*κ* = 0.45).

## Discussion

A classification should have good validity and reproducibility [11]. The reproducibility depends on inter- and intra-observer agreement. The mean kappa value for inter-observer agreement of the main types (A, B and C) and that of its groups (A_2-3_, B_1-3_ and C_1-3_) were both found to be moderate but with a good kappa value for the intra-observer observer agreement. For the trauma surgeon group in particular, the mean kappa value of the inter-observer agreement was moderate in both main types and groups in combination with a moderate (types) and good (groups) intra-observer agreement.

For the exclusive C-type fracture group, the mean kappa coefficient of the inter-observer agreement for groups (C_1-3_) was fair, with a moderate intra-observer agreement for all observers and for the consultant trauma surgeons in particular.

### Previous literature

The results of this study of the inter-observer agreement of the eight groups (*κ* = 0.48) were comparable with the results of Kreder et al. [7] (SAV = 0.48). The SAV value is a kappa value for multiple assessors. Other studies which date from 1996 and 2001 showed a lower agreement: Both studies recorded a kappa of 0.30 [6, 8]. However, more recent studies showed comparable results: After reviewing 98 cases in 2008, Belotti et al. concluded that the inter-observer agreement was moderate (*κ* = 0.49) in the AO/ASIF classification system [14]. In 2015, Plant et al. classified 456 patients and also found a moderate (*κ* = 0.56) inter-observer agreement for AO types. They concluded that inclusion of groups and subtypes reduced the agreement to fair (*κ* = 0.29 and 0.28) [15]. However, in our study the addition of groups to the type of fracture did not show any significant decrease in the mean kappa value (*κ* = 0.49 resp. *κ* = 0.48). This might be explained by the fact that we used both pre- and post-reduction radiographs yielding more detailed information at the group level.

The result from our study (*κ* = 0.49) is at the lower end of the ‘moderate’ spectrum. Andersen, Oskam, and Kreder found higher kappa values, respectively, 0.64, 0.68 (SAV), and 0.65 [6, 7, 9]. Only the study of MacDermid showed a lower kappa value (*κ* = 0.35) [8]. The inter-observer agreement of C-type fractures in our study at group level (*κ* = 0.27) approaches the agreement found by Illarramendi (*κ* = 0.37) [16] and is considered to be too low for reliable prognostic evaluation, research purposes, or fracture planning management.

We included only patients with pre- and post-reduction radiographs in contrast to the study of MacDermid [8]. Where available, pre- and post-reduction radiographs were used in the study of Andersen [6]. The availability of two radiograph series instead of only one could very well have led to a higher kappa coefficient. However, since reduction is commonly performed before surgery, it was deemed appropriate in our present study to include post-reduction radiographs for the assessments as well. Also, in contrast to our study, Andersen excluded radiographs of poor quality. Poorer-quality radiographs are more difficult to classify and could have led to a lower kappa coefficient in our study [6]. A higher agreement in the study of Oskam et al. might have been caused by the fact that fractures that could not be attributed to a particular AO main group (ABC) were classified as type D. Therefore, a separate category for undisplaced distal radius fractures in the AO classification was recommended by them [9].

Illarramendi et al classified distal radius fractures in five categories: group I included AO type A fracture, group II included AO type B fractures, group III, IV, and V were type C1, C2, and C3 fractures, respectively [14]. The inter-observer agreement in this study was *κ* = 0.37 (0.25–0.48). Their classification into two main groups A and B and the three subtypes for C fractures might be an explanation for their higher inter-observer kappa value compared to our study since the agreement of the three main groups in our study is also higher (*κ* = 0.49) than the agreement of C-type fractures. Another explanation is that the radiographs that were not classified in one of the pre-specified groups of fractures were excluded by Illarramendi et al.

### Limitations

While the kappa values were calculated by marginals, reasonable agreement could possibly have resulted in a low kappa value if the marginals contained small amounts. Since our study population contained relatively few type B fractures, this might have resulted in a skewed distribution and therefore a lower kappa value [11]. In order to prevent assessment bias, clinical information was not available for the observers despite the fact that all patients had been operatively treated. However, this patient-related information is of great importance on decision-making in daily practice, and it could be argued that patient information should have been added. However, our aim was to assess the reproducibility of radiograph interpretation as ‘lean’ as possible, and therefore, patient details were left out.

Also, fracture analysis could have been complicated in the post-reduction series by the applied cast although this was considered the most realistic method since it exactly resembles clinical practice. Moreover, the initial radiograph series showed unreduced fractures without a cast. Knowing that all patients were treated operatively, severity of the fracture might be overrated by the observers leading to bias.

In conclusion, the overall inter-observer agreement of the main AO types and their groups was moderate with good intra-observer agreement. Among the consultant trauma surgeons, the inter-observer agreement was fair with moderate intra-observer agreement for the main types and good intra-observer agreement for the groups. For C-type fractures in particular, the overall inter-observer agreement was fair with moderate intra-observer agreement. These results show that the AO classification for distal radius fractures requiring operative treatment does not have an adequate reproducibility. Classification of distal radius fractures with both pre- and post-reduction radiographs might lead to a higher inter-observer agreement although the agreement is still not sufficient. A simplified classification system may improve agreement among clinicians.
